# Whole-genome sequence data of cellulase-producing fungi *Trichoderma asperellum* PK1J2, isolated from palm empty fruit bunch in Riau, Indonesia

**DOI:** 10.1016/j.dib.2022.108607

**Published:** 2022-09-15

**Authors:** Fela Laila Nur Hidayati, Dian Anggraini Suroto, Sardjono, Muhammad Nur Cahyanto, Jaka Widada

**Affiliations:** a Department of Food and Agricultural Product Technology, Faculty of Agricultural Technology, Universitas Gadjah Mada, Jl Flora, Bulaksumur, Yogyakarta 55281, Indonesia; b Department of Agricultural Microbiology, Faculty of Agriculture, Universitas Gadjah Mada, Jl. Flora, Bulaksumur, Yogyakarta 55281, Indonesia

**Keywords:** Whole-genome sequence, *Trichoderma asperellum*, Palm empty fruit bunch, Genomic

## Abstract

*Trichoderma asperellum* PK1J2 is a promising cellulase-producing fungus isolated from a palm empty fruit bunch in Riau, Indonesia. Presented here is the genome assembly of *T. asperellum* PK1J2. The whole genome of the fungi was sequenced using Illumina NovaSeq PE150. The genome assembly was performed using SOAPdenovo, SPAdes, and Abyss software, and the assembly results of the three types of software were integrated with CISA software. *T. asperellum* PK1J2 has 6,835 protein-coding genes with a length of 9,233,597 bp. The final genome assembly was approximately 36 Mbp with a GC content of 48.45%. This whole genome shotgun project has been deposited at DDBJ/ENA/GenBank under accession JAGJIK000000000.

**Specifications Table**

**Table:** 

Subject	Biological science
Specific subject area	Genomics, Microbiology
Type of data	Genome sequencing in FASTA format Table Figure
How the data were acquired	Genome sequencing was performed using Illumina Novaseq PE150
Data format	Raw Analyzed
Description of data collection	Genomic DNA was isolated from *Trichoderma asperellum* PK1J2. The sequencing libraries were generated using the NEBNext® ULtra™ DNA Prep Kit for Illumina (NEB, USA). Illumina NovaSeq PE150 was used for whole genome sequencing. The genome assembly was performed with SOAPdenovo, SPAdes and Abyss software and integrated with CISA software.
Data source location	Institution: Faculty of Agricultural Technology, Universitas Gadjah Mada City/Town/Region: Sleman, Yogyakarta Country: Indonesia Latitude and longitude for samples/data collection: 7° 46′ 14.5″ S, 110° 22′ 39.8″ E
Data accessibility	Repository name: Data identification number: This whole genome shotgun project has been deposited at DDBJ/ENA/GenBank under accession JAGJIK000000000. The version described in this paper is version JAGJIK000000000. Direct URL to data: www.ncbi.nlm.nih.gov/assembly/GCA_022817925.1/ The raw sequence data of this paper are accessible under SRA accession number SRR19762116. Direct URL to data: www.ncbi.nlm.nih.gov/sra/?term=pk1j2 All data in this paper are available at NCBI with BioProject number PRJNA699105. Direct URL to data: www.ncbi.nlm.nih.gov/bioproject/PRJNA699105

## Value of the Data

• The genome data of *Trichoderma asperellum* PK1J2 isolated from Indonesia provide insight into the genetic diversity of *T. asperellum* and essential genetic information to reveal important details of effector proteins, metabolites and enzymes production.
• The data can be useful for researchers working on fungal microbiology, biotechnology, genomics, and genetic engineering.
• This genome information can be used for genome mining to discover the genes involved in metabolites and enzymes biosynthesis pathways.
• Stakeholders, including industry, can use *T. asperellum* PK1J2 as a biocontrol agent, biofertiliser, and producer of metabolites and enzymes, especially cellulase, through this genetic information.

## 1 Data Description

*T. asperellum* is a mycoparasitic species widely used for its ability to inhibit the growth of plant pathogens [1]. *T. asperellum* has been shown to produce hydrolytic enzymes such as cellulase and xylanase [2,3]. *T. asperellum* has also been reported to hydrolyse wheat bran, wheat straw, paper, sawdust, corncob, duckweed, and agave by secreting cellulases [2], [3], [4], [5], [6], [7]. Strain PK1J2 has been proven to be capable of producing high cellulase. The cellulase from this fungi can hydrolyse cassava stem and sago waste into fermentable sugar [8,9]. In a previous study, strain PK1J2 produced highest cellulase activity among the examined fungi isolated from Indonesia and was further selected to characterize its genome.

**Fig. 1:**
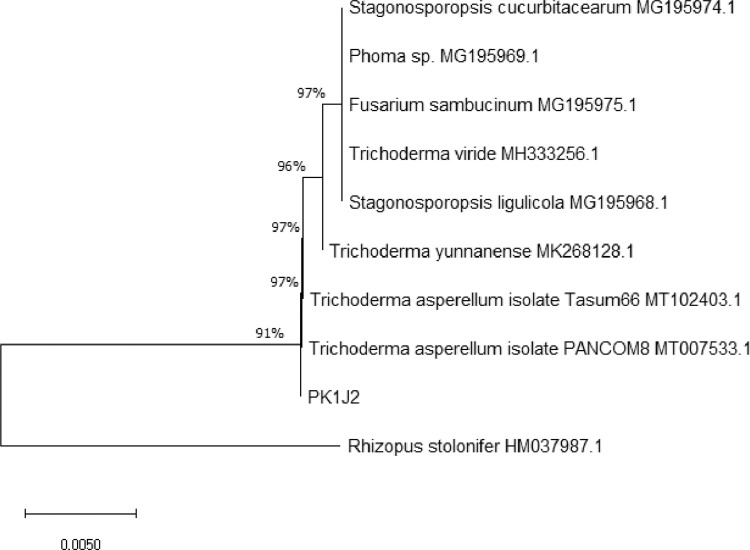
Phylogenetic tree of strain PK1J2 ITS region compared with the other fungi.

Fig. 1 shows a phylogenetic tree of strain PK1J2 comparing its internal transcribed spacer (ITS) region with the other fungi. As can be seen in the figure, the ITS gene of strain PK1J2 showed the highest similarity with *Trichoderma asperellum* species.

*T. asperellum* PK1J2 had 6835 protein-coding genes with 9,233,597 bp in length, as seen in Table 1. The final assembly for the *T. asperellum* PK1J2 genome was approximately 36 Mbp with a GC content of 48.45%. The genome consisted of 249 scaffolds with a total length of 36,156,613 bp (N_50_, 724,251 bp; N_90_, 188,633 bp; and N_max_, 2,963,926 bp) and 636 contigs with a total length of 36,152,186 bp (N_50_, 143,266 bp; N_90_, 33,810 bp; and N_max_, 506,821 bp). This whole genome shotgun project has been deposited at DDBJ/ENA/GenBank under accession JAGJIK000000000. The version described in this paper is version JAGJIK000000000 [10].

**Table 1:** Genome features of *T. asperellum* PK1J2.

Genome feature	Value
Genome size	36,156,613
Gene number	6835
Gene length	9,233,597
tRNA genes	236
rRNA genes (5 s)	49
sRNA genes	2
snRNA genes	22

Functional gene annotation predicted about 4759 genes using GO, 6398 genes using KEGG, 1946 genes using KOG, 4759 genes using Pfam, 2783 genes using SWISS-PROT, and 6544 genes using NR database. Gene coding for protein possibly involved in secondary metabolite production revealed the presence of T1PKS cluster, NRPS cluster, NRPS-like cluster, T1PKS-NRPS hybrid cluster, and terpene cluster. A carbohydrate-active enzyme analysis showed that *T. asperellum* PK1J2 was dominated by GH18, GH3, GH16, GH2, and GH5.

## 2 Experimental Design, Materials and Methods

### 2.1 Fungal Strain and DNA Extraction

Strain *Trichoderma asperellum* PK1J2 was obtained from the Laboratory of Biotechnology, Faculty of Agricultural Technology, Universitas Gadjah Mada. Strain PK1J2 was isolated from a rotten palm empty fruit bunch, Pekanbaru, Riau, Indonesia. The strain was grown on PDA agar at 30 °C for a period of seven days. ZymoBIOIMICS™ DNA Mini Kit (Zymo Research, California) was used for extracting genomic DNA. The harvested DNA was detected by agarose gel electrophoresis and quantified by Qubit® 2.0 Fluorometer.

### 2.2 Species Identification

The DNA fragment was amplified using universal primer set ITS1 (forward primer) 5′-TCCGTAGGTGAACCTGCGG-3′ and ITS4 (reverse primer) 5′-TCCTCCGCTTATTGATATGC-3′. The PCR product was sequenced using Bi-directional Sequencing. The sequence was analyzed by BLAST and then compared to the NCBI database. The phylogenetic tree was constructed using the Neighbor-Joining method (Unrooted Tree) by NCBI BLAST.

### 2.3 Genome Sequencing and Assembly

Sequencing libraries were generated using NEBNext® Ultra™ DNA Library Prep Kit for Illumina (NEB, USA) following manufacturer's recommendations. The whole genome sequencing of the fungi was performed using an Illumina NovaSeq PE150 at the Beijing Novogene Bioinformatics Technology Co., Ltd. The genome assembly was done using SOAPdenovo, SPAdes, and Abyss software. The assembly results from all three software were integrated with CISA software. The assembly result with the least scaffolds was selected.

### 2.4 Genome Component Prediction

Transfer RNA (tRNA) genes were predicted by tRNAscan-SE [11]. Also, ribosome RNA (rRNA) genes were analyzed by rRNAmmer [12], and small nuclear RNAs (snRNA) were predicted by BLAST against the Rfam database [13].

### 2.5 Genome Annotation

Genome functional annotation was based on the BLASTP with GO (Gene Ontology) [14], KEGG (Kyoto Encyclopedia of Genes and Genomes) [15], COG (Clusters of Orthologous Groups) [16], NR (Non-Redundant Protein Database) [17], and SWISS-PROT [18]. Carbohydrate-active enzymes were predicted by the Carbohydrate-Active Enzymes Database (CAZy) [19]. Genes coding for proteins that were possibly involved in secondary metabolite production were predicted by antiSMASH v.5.0 [20].

## Ethics Statements

Not applicable.

## CRediT authorship contribution statement

**Fela Laila Nur Hidayati:** Investigation, Formal analysis, Writing – original draft. **Dian Anggraini Suroto:** Methodology, Software, Data curation. **:** Resources. **Muhammad Nur Cahyanto:** Supervision, Project administration, Funding acquisition, Writing – review & editing. **Jaka Widada:** Conceptualization, Methodology, Validation, Writing – review & editing.

## Declaration of Competing Interest

The authors declare that they have no known competing financial interests or personal relationships that could have appeared to influence the work reported in this paper.

## Data Availability

richoderma asperellum strain:PK1J2 (Original data) (National Center for Biotechnology Information (NCBI)).
